# Aromatic sensitizers in luminescent hybrid films†

**DOI:** 10.1039/d2ra03360g

**Published:** 2022-06-20

**Authors:** Per-Anders Hansen, Joachim Svendsen, Hanne Nesteng, Ola Nilsen

**Affiliations:** Department of Chemistry, Centre for Materials Science and Nanotechnology, University of Oslo Sem Sælandsvei 26 0371 Oslo Norway p.a.hansen@kjemi.uio.no

## Abstract

Atomic layer deposition offers a unique set of design possibilities due to the vast range of metal and organic precursors that can be used and combined. In this work, we have combined lanthanides with aromatic aids as strongly absorbing sensitizers to form highly luminescent thin films. Terephthalic acid is used as a base sensitizer, absorbing shorter wavelengths than 300 nm. The absorption range is extended towards the near-UV and blue range by increasing the aromatic system and adding functional groups that have strong red-shifting effects. While terbium and europium provide green and red emission, yttrium allows emission from the sensitizer itself spanning the whole color range from purple, blue and green to red. Many organic dye molecules show very high luminescence quantum yields and several of the molecules and materials investigated in this work show bright luminescence.

## Abstract 2

By combining Y^3+^, Eu^3+^ and Tb^3+^ with a range of aromatic acids in molecular layer deposition, we achieve strong near-UV absorption and colorful luminescence.

## Graphical abstract

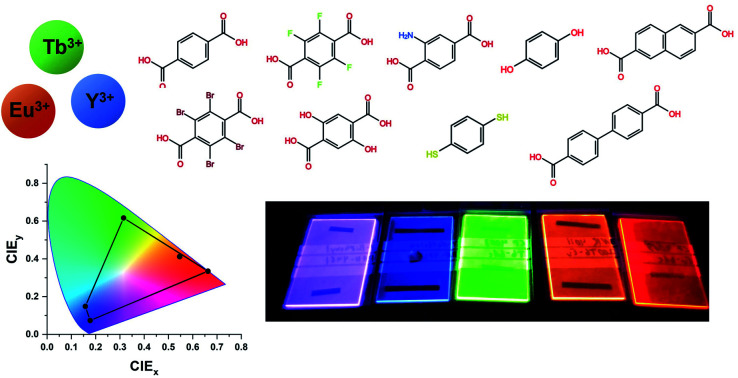

## Introduction

Atomic layer deposition (ALD) and its organic counterpart molecular layer deposition (MLD) is a remarkable synthesis tool capable of synthesizing a vast array of materials ranging from inorganic^1^ including quaternary^2^ and even quinary compounds,^3^ nanostructures,^4^ over to organic–inorganic hybrids,^5^ crystalline MOF films^6^ and pure organics.^7^ The layer-by-layer chemistry and low deposition temperatures prevents interdiffusion between layers and enables sub-nanometer scale designs, while the vast array of deposition materials allows combinations and layered nanocomposites of different material classes. We have recently shown how combining ALD and MLD allows designing nanocomposites that combines luminescence properties of inorganic fluorides and optical absorption of organic molecules,^8^ in addition to controlling and confining movement of excited states and thus reducing concentration quenching effects.^9^ In addition, deposition by ALD allows on high aspect ration 3D nanostructures like photonic crystals or waveguides, and the (generally) low deposition temperature can be compatible with temperature sensitive substrate materials like textiles and polymers.

The inclusion of organic species into hybrid or nanocomposite thin film is an exciting field as the organic component adds very strong optical absorption and an exceptional flexibility in designing the molecules towards specific absorption and emission ranges. Unfortunately, the choice of organic components in MLD is quite limited. The need to get these molecules into the vapour phase while also having sufficient reactivity reduces the practical range to fairly small molecules. This puts strong limits on the range of optical and electronic properties to choose from.

Reviews on ALD and MLD chemistries^7,10^ reveals that the organic precursors utilized so far are quite limited beyond a single aromatic ring or a short conjugated chain. There are examples of larger conjugated systems like naphthalene and biphenyl^6^ showing near-UV activity and even visible absorption.^11^ These optically absorbing dye precursors require sublimation temperatures of around 250 °C or more, approaching the temperature where such organic molecules start to decompose or polymerize. This illustrates well the difficulty in balancing Vis and NIR active optical properties with ALD/MLD compatibility. An exception to this is our recent work on quinizarin molecules, which sublimes efficiently at 130 °C and produces deep pink films with TMA as cation precursor.^12^

**Fig. 1:**
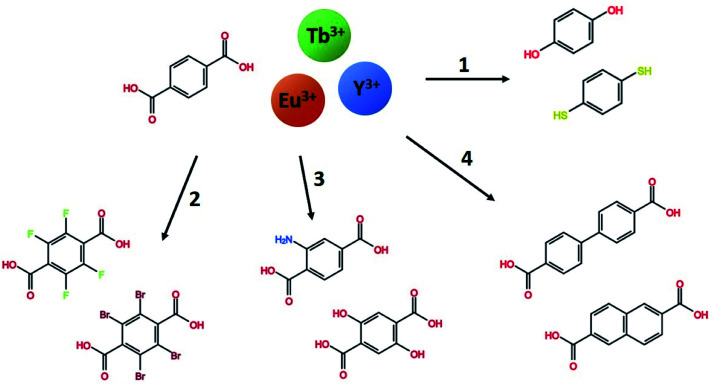
The range of mono- and bi-aromatic di-carboxylic acids investigated in this work, combined with the three lanthanides Tb, Eu and Y. Using terephthalic acid and Tb as a base system, four paths of molecule variations are explored. (1) Other binding group in H_2_bdo and H_2_bdt, (2) halogenated H_2_bdc-F and H_2_bdc-Br, (3) functionalized H_2_bdc-NH_2_ and H_2_bdc-2OH, and (4) the larger conjugated system H_2_bpdc and H_2_ndc.

In this work, we explore a range of mono- and bi-aromatic di-carboxylic acids deposited with lanthanides as cations, as shown in Fig. 1. All three lanthanides have very similar deposition chemistries.^13^ Terbium and europium was chosen due to their well-known and easily identified sharp emission peaks which are easily distinguishable from organic molecule emissions. In addition, these two lanthanides have large energy gaps from the emitting energy level(s) to the next lower energy level. This allows them to be highly luminescent even in high-phonon matrices such has hybrid materials. Yttrium was chosen as an optically inactive ion that allows characterization of the organic molecules emission properties in the same lanthanide-organic hybrid material environment. In previous works, we have investigated the growth and material chemistry of optically active hybrid materials.^5,6,12,14^ Here, we focus on the hybrid materials optical properties, absorption and luminescence colour.

## Experimental

The films were deposited in an F-120 research-type ALD-reactor (ASM Microchemistry Ltd). The deposition temperature were 250 °C except when otherwise noted. Ln-β-diketonate Ln(thd)_3_ (Strem Chemicals, >99.9%, Ln = Y, Eu, Tb) were used as lanthanide precursors, while a range of different organic precursors were used as optical sensitizers. The full names, abbreviations used in this work and the sublimation temperatures used for all precursors are listed in Table 1. Nitrogen was used as carrier and purge gas, supplied from gas bottles (AGA, 99.999%). p-Type Si(100) substrates were used for all depositions. In addition, 0.5 × 4 cm^2^ Si(100) strips were placed some 8 cm apart in the gas inlet and exhaust sides of the deposition chamber to monitor thickness gradients. Silicon substrates were used with native oxide intact and its thickness was measured before each deposition. All substrates were dry wiped and dust was removed using pressurized air. Pulse times for Ln(thd)_3_ and organics were 1.5 and 2 seconds respectively. Purge times were between 1 and 2 seconds. Exceptions to the standard parameters are that H_2_bpdc was deposited at 275 °C due to its high precursor temperature, H_2_bdt was deposited at 200, 250 and 300 °C, and H_2_bto depositions used 3 second lanthanide and organic pulses. In addition, Tb_2_bdc_3_ was used as a basis material and was deposited in the 225–350 °C range in steps of 25 °C.

**Table 1:** Precursor names and sublimation temperatures

	Compound name	Short name	*T*_sub_ (°C)
	Ln(2,2,6,6-tetramethyl-3,5-heptanedione)_3_	Y(thd)_3_	130
		Eu(thd)_3_	150
		Tb(thd)_3_	145
	1,4-Benzenedicarboxylic acid	H_2_bdc	200
(1)	2,3,5,6-Tetrafluoro-1,4-benzenedicarboxylic acid	H_2_bdc-F	145
	2,3,5,6-Tetrabromo-1,4-benzenedicarboxylic acid	H_2_bdc-Br	230
(2)	2-Amino-1,4-benzenedicarboxylic acid	H_2_bdc-NH_2_	210
	2,5-Dihydroxy-1,4-benzenedicarboxylic acid	H_2_bdc-2OH	225
(3)	Biphenyl-4,4′-dicarboxylic acid	H_2_bpdc	250
	2,6-Naphthalenedicarboxylic acid	H_2_ndc	225
(4)	Benzenediol	H_2_bdo	130
	Benzenedithiol	H_2_bdt	70

*In situ* quartz crystal microbalance (QCM) analyses were conducted using a 6 MHz AT-cut quartz crystal. The crystal was mounted in a home-made holder and was used to monitor the mass increase, proportional to the change in frequency during the deposition to determine saturation conditions for pulse and purge parameters. The signal was recorded using a Colnatec Eon-LT and processed by averaging over 16 consecutive ALD cycles. The temperature was stabilized for 60 to 90 minutes before any experiments were conducted to ensure a stable temperature and response from the QCM-crystals. Note that the QCM investigations were conducted over an extended timeframe where modifications to the setup have occurred. Thus, the scale of the frequency changes is not necessarily comparable between different materials in this work.

Film thickness and refractive index *n*(*λ*) were determined with a J. A. Woollam alpha-SE spectroscopic ellipsometer in the 380–900 nm range. This ellipsometry data was modelled using a Cauchy model. A VASE spectroscopic ellipsometer with range of 280–1000 nm, also from J. A. Woollam, was used to model *n*(*λ*) and the extinction coefficient *k*(*λ*). These films were modelled with a general oscillator model consisting of several Gaussian peaks. Silicon samples were used for ellipsometry. In both instruments, the sample is modelled by a single layer on top of a thin native silicon oxide layer. This native oxide was measured before each deposition and was generally in the 2–4 nm range. Interface and surface roughnesses were not included in the model as these did not improve the fit. The relation between absorption coefficient *α*(*λ*) and *k*(*λ*) is given in eqn (1). For readers more accustomed to *α*(*λ*), the practical difference between these is a constant scaling factor (2*π*) a simple wavelength dependent factor (1/*λ*). FTIR characterization was done using a Bruker VERTEX 70 FTIR spectrometer in transmission mode on single-side polished silicon substrates. Luminescence (PL) and excitation (PLE) measurements was done using an Edinburgh Instruments FLS920 fluorescence spectrometer with a 450 W Xe lamp as excitation source and a Hamamatsu R928 PMT for detection. PL decay measurements utilized an optical parametric oscillator (OPO) system (Opotek HE 355 II) pumped by the third harmonic of a Nd:YAG laser as excitation source. The OPO system was set to 355 nm and a repetition rate of 20 Hz. The detector for decay measurements was the same equipment used for the PLE.1
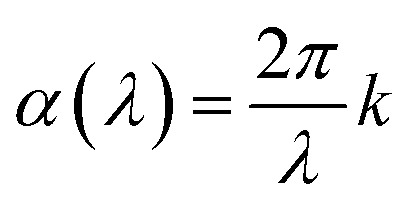


## Results and discussion

This work uses Tb_2_bdc_3_ as basis material, modifying the luminescence and absorption properties by replacing Tb^3+^ with other lanthanides and bdc with other aromatic molecules. Thus, the growth and properties of Tb_2_bdc_3_ was investigated in more detail than the other materials, while the other materials focus on their differences compared to Tb_2_bdc_3_.

### Tb_2_bdc_3_ – growth and optical properties

**Fig. 2:**
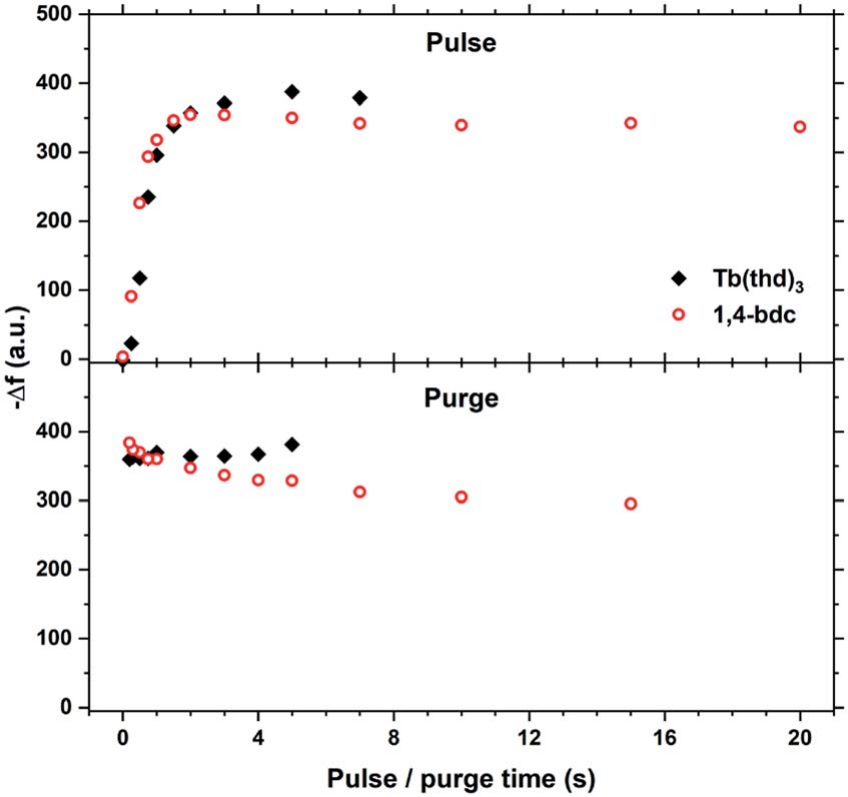
QCM characterization of growth saturation as a function pulse durations for Tb_2_bdc_3_.

The saturation conditions for Tb_2_bdc_3_ based on QCM experiments is given in Fig. 2. Both lanthanide and acid precursor show clear saturation at around 2 seconds pulses. Purge durations have little effect apart from a gradual growth rate reduction upon long acid purges.

**Fig. 3:**
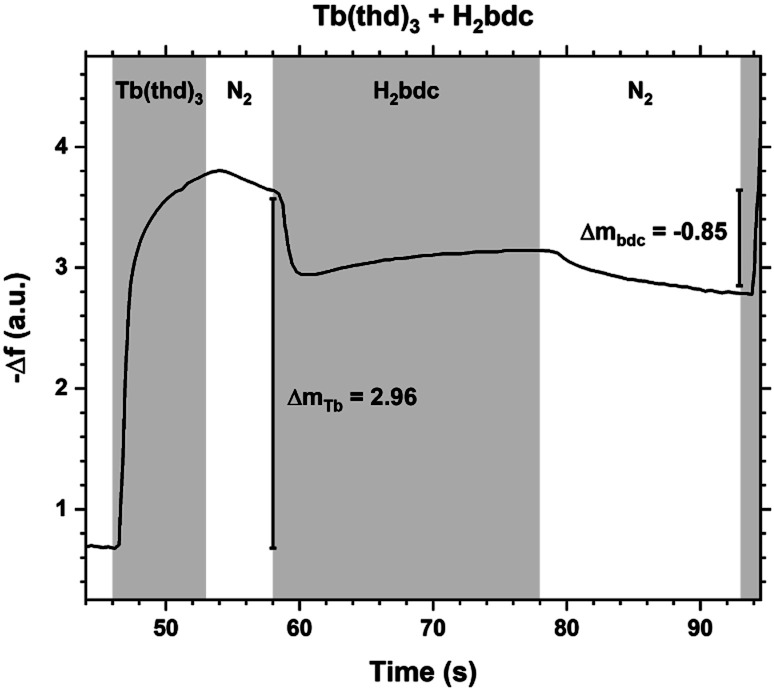
QCM response during a Tb_2_bdc_3_ cycle with long pulse and purge times. Tb(thd)_3_/purge/1,4-bdc/purge = 7/5/20/15.

The QCM response during a cycle with long pulse and purge times is shown in Fig. 3. The two ideal half cycle reactions are given below. For comparison, the masses of the precursors and leaving groups are: Tb(thd)_3_ = 709, H_2_bdc = 166, Hthd = 184 g mol^−1^. Both the lanthanide and acid pulses show initial rapid frequency change of around 2 seconds followed by a slower evolution. The mass decrease during the acid pulse is due to H_2_bdc being slightly lighter than the leaving molecule Hthd. The ratio between the ideal reactions are Δ*m*_1_/Δ*m*_2_ = 16, while the measured Δ*m*_Tb_/Δ*m*_bdc_ = 3.5. This indicate that either less lanthanide or more acid is deposited during each half cycle than expected from the ideal reactions.2
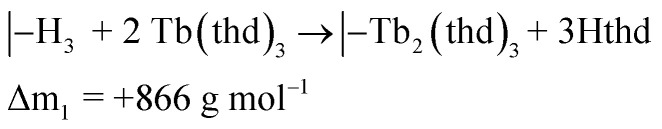
3
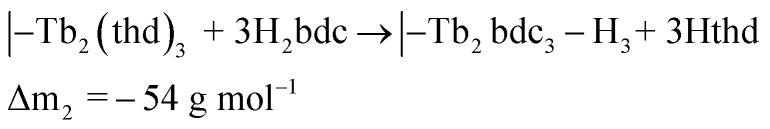


**Fig. 4:**
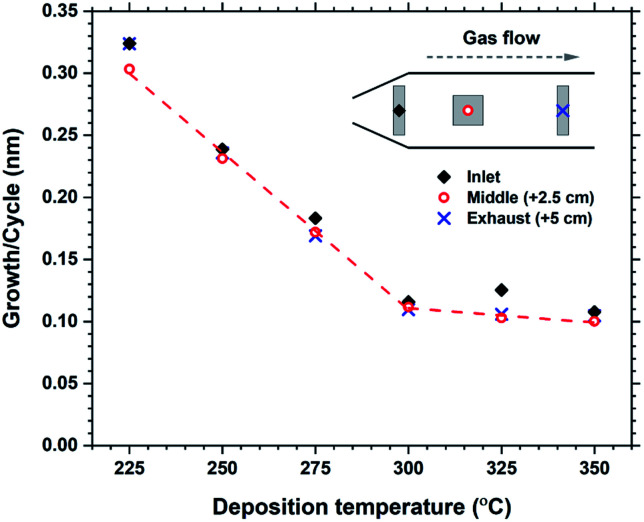
Growth rate as a function of deposition temperature. Measurements from 3 different locations over a 5 cm distance indicates the evenness at each temperature.

The growth rate as a function of deposition temperature is shown in Fig. 4. The growth decreases linearly with temperature up until 300 °C, and becomes almost stable up to 350 °C. The films above 300 °C had a brown-ish colour and was non-luminescent indicating thermal decomposition of either H_2_bdc or Hthd, or both. The luminescence intensity of the films deposited in the 225–275 °C range did not vary significantly. Thus it was decided to keep the deposition temperature at 250 °C for all films, unless otherwise stated.

**Fig. 5:**
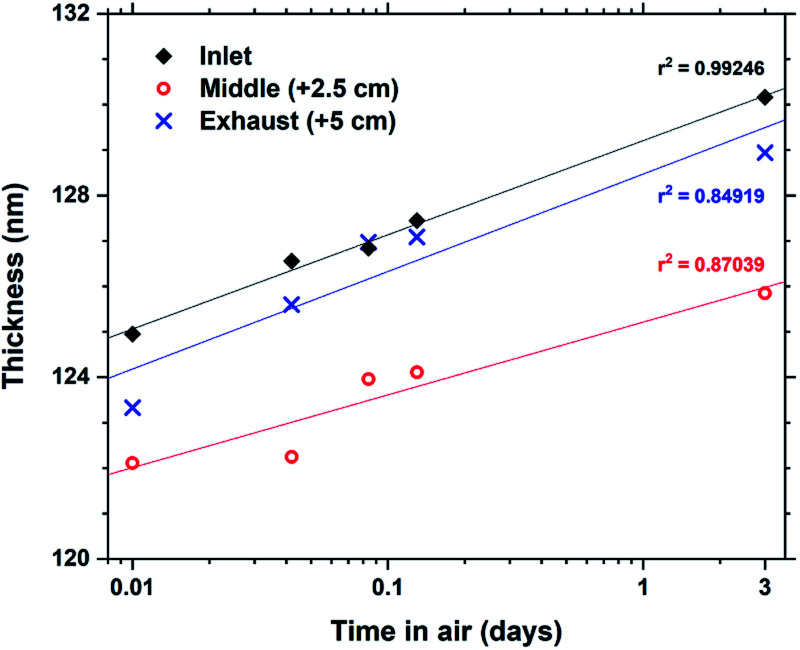
Evolution of film thickness upon air exposure for Tb_2_bdc_3_ films.

Some hybrid materials are not long-term stable in air, either through absorption and release of for example moisture or oxidation reactions. Tb_2_bdc_3_ films were characterized by ellipsometry immediately after deposition and 4 additional times up to 3 days to monitor any changes to the films thickness. The results are shown in Fig. 5. The films show a swelling of about 4% after 3 days with no other apparent changes to the films structure or properties. In fact, films deposited over 4 years ago show identical optical and luminescent properties as freshly deposited films.

**Fig. 6:**
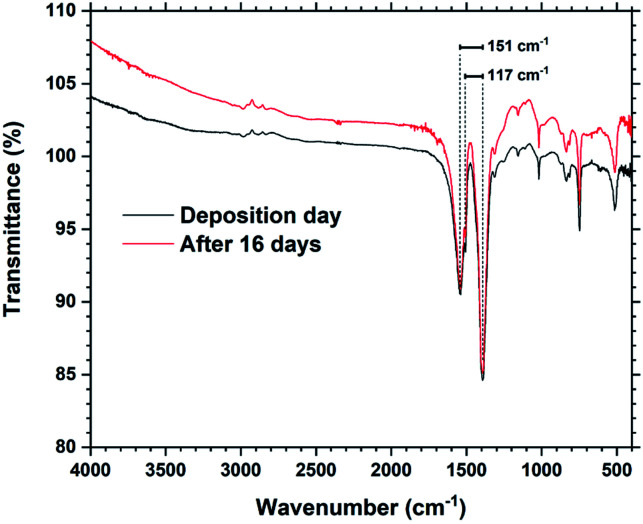
FTIR spectra of a Tb_2_bdc_3_ film taken on the day of deposition and after 16 days. The splitting between the asymmetric and symmetric peaks are shown.

Fig. 6 shows FTIR spectra from a Tb_2_bdc_3_ film taken during the day of deposition and 16 days later. The spectra show that there are no significant differences in number, position or intensities of peaks, indicating no change in bonding during this time. The films show no sign of protonated carboxylic acid groups, which should be clearly visible around 3000 cm^−1^. FTIR is useful to investigate the type of bonding between the metal and acid group based on the splitting (*Δ*_acid_) between the asymmetric and symmetric carboxylate stretching peaks.^15^ A *Δ*_acid_ in the 50–150 cm^−1^ range indicate bidentate binding, 130–200 cm^−1^ indicate bridging and >200 cm^−1^ indicate unidentate binding. The asymmetric carboxylic stretching peak is also split into a double peak, giving two values for *Δ*_acid_, 151 and 117 cm^−1^. This indicates that the Tb_2_bdc_3_ films contains a mixture of bidentate and bridging binding. An enlarged version zoomed in on these peaks is shown in ESI Fig. 1,† where also the exact wavenumber position of these three peaks are numbered.

**Fig. 7:**
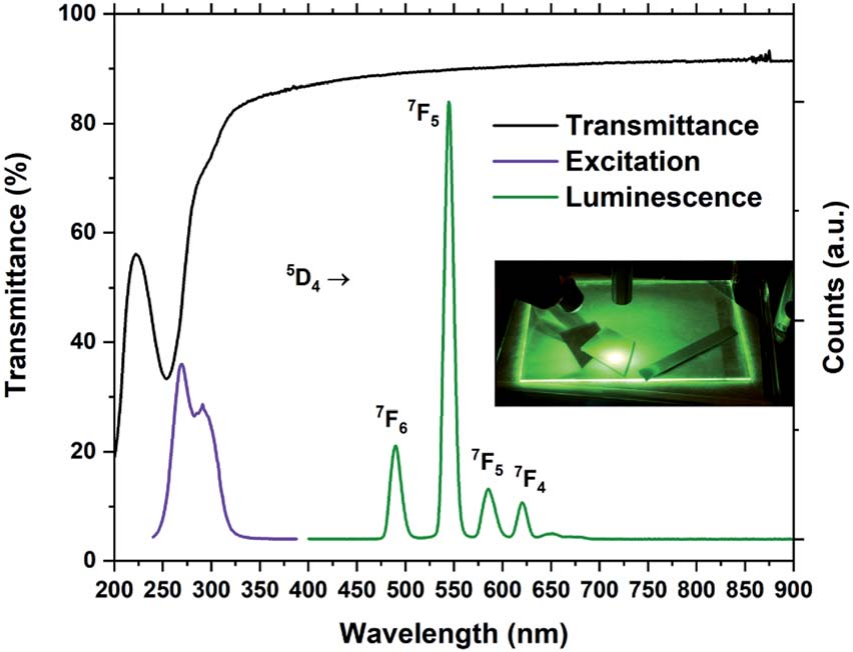
Transmittance, PL and PLE spectra of Tb_2_bdc_3_. The inset shows a glass plate and silicon substrates coated with Tb_2_bdc_3_ under 280 nm illumination from a diode.

The optical absorption and luminescence properties of Tb_2_bdc_3_ is shown in Fig. 7, alongside a photograph of a sample under a 280 nm diode. Transmittance data is obtained with a 28 nm film on silica substrate, where the multiplet absorption from the benzene ring of terephthalic acid is clearly seen at around 250 and 300 nm. The PLE spectrum is obtained by monitoring the ^5^D_4_ → ^7^F_5_ emission. The two first aromatic absorption peaks are clearly seen in the PLE spectrum. The spectrum is not corrected for the decrease in the Xe lamps light intensity towards deep UV, which causes the apparent red-shift of the high energy peak.

Fig. 7 forms the basis for exploring the other organic sensitizers and lanthanides with the aim to obtain absorption towards the near-UV and blue range and different emission colours. Each of the 4 groups of molecules illustrated in Fig. 1 is compared to Tb_2_bdc_3_ individually below, while the deposition parameters and obtained growth rates are summarized in Table 2.

**Table 2:** Summary of growth rates with Tb(thd)_3_

	Organic	*T*_dep_ (°C)	*g* (nm/cycle)	Parameters (s)
	bdc	250	0.23	1.5/1.5/2/1
(1)	bdo	175	0.12	3/1/3/1
	bdt	250	0	1.5/1.5/2/1
(2)	bdc-F	250	0.24	1.5/1.5/2/1
	bdc-Br	250	0.27	1.5/1.5/2/1
(3)	bdc-NH_2_	250	0.27	1.5/1.5/2/1
	bdc-2OH	250	∼1^a^	1.5/1.5/2/2
(4)	bpdc	275	0.26	1.5/1/2/1
	ndc	250	0.27	1.5/1.5/2/2

a All films showed strong gradients.

### 1,4-Benzenediol and 1,4-benzenedithiol

The aim of these two R–OH and R–SH molecules is to investigate if other binding groups that carboxylic acid can be used to obtain hybrid films with Ln(thd)_3_, in addition to how these bonds affect the materials optical properties. In particular the sulfur atom can participate in low energy charge transfer transitions and lower the d-shell and create low energy f → d transitions with lanthanides.^16^

Films deposited with Tb(thd)_3_ and H_2_bdo showed a growth rate of 0.13 nm per cycle, which is about half of the growth rate of Tb_2_bdc_3_. The films were visually transparent like Tb_2_bdc_3_, however these films did not luminesce under a 254 nm UV lamp. As enhancing the optical and luminescent properties of lanthanide-hybrid materials is the aim of this work, the H_2_bdo precursor was not further explored apart from noting that H_2_bdo and Tb(thd)_3_ do react and form ALD films. Thus, hydroxy groups can be suitable reaction groups to bind to Ln(thd)_3_. In fact, H_2_bdo sublimes at 70 °C lower than H_2_bdc, which can be a desired property.

Tb(thd)_3_ and H_2_bdt did not result in film deposition. Film growth was attempted at 200, 250 and 300 °C. This was somewhat surprising as H_2_S can be used with Ln(thd)_3_ to produce lanthanide sulfide films.^17^

### Tb_2_bdc_3_: Ln^3+^ and halogenated bdc

Halogenation of the aromatic centre of H_2_bdc can affect both the growth and optical properties in addition the physical properties of the precursor. From Table 1 it is seen that H_2_bdc-F and H_2_bdc-Br sublimes at 70 °C lower and 30 °C higher than H_2_bdc, respectively. Table 2 show that both precursors result in similar growth rates as H_2_bdc. Regarding growth, the main difference between H_2_bdc, H_2_bdc-F and H_2_bdc-Br is a substantial difference in sublimation temperature.

**Fig. 8:**
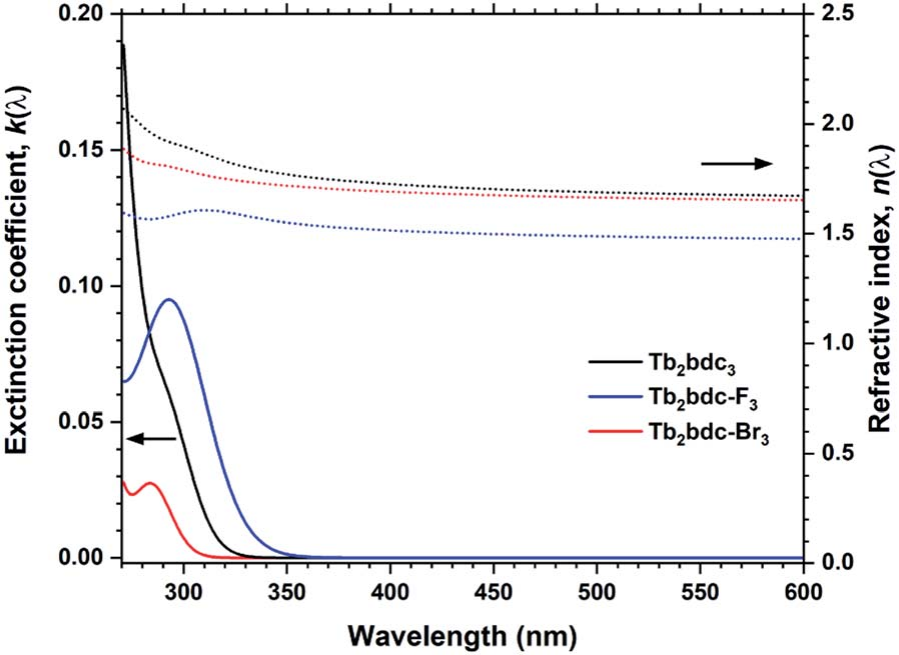
*k*(*λ*) and *n*(*λ*) for Tb_2_bdc_3_, Tb_2_bdc-F_3_ and Tb_2_bdc-Br_3_ films.

**Fig. 9:**
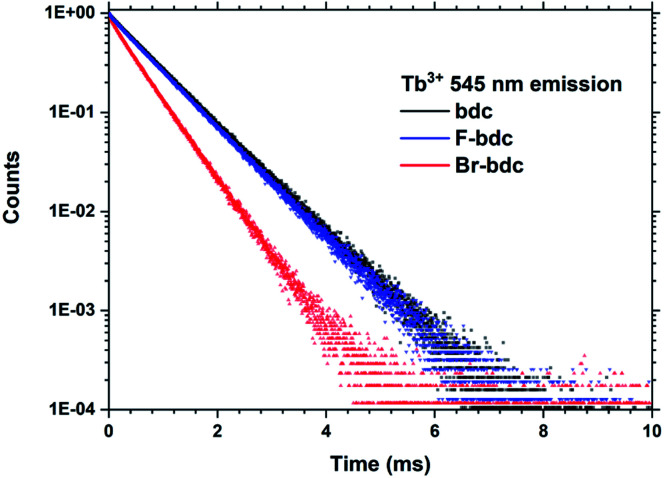
PL decay data from Tb_2_bdc_3_, Tb_2_bdc-F_3_ and Tb_2_bdc-Br_3_ films.

Films produced with these three precursors and Tb(thd)_3_ all produced luminescent with the same green colour and with similar intensity under a 254 nm UV lamp. To further investigate differences in optical properties, the films optical absorption and luminescence decays were characterized. *k*(*λ*) and *n*(*λ*) of these films is shown in Fig. 8. Compared to Tb_2_bdc_3_, fluorination causes a red-shift in absorption and a lower *n*(*λ*) while bromination causes a blue-shift with unchanged *n*(*λ*). Fig. 9 show decays of the ^5^D_4_ → ^7^F_5_ emissions of the three materials. All three can be fitted with a single exponential model. The brominated sample show a lower lifetime than Tb_2_bdc_3_ and Tb_2_bdc-F_3_. This could indicate higher quenching rates in Tb_2_bdc-Br_3_. In summary, bromination offers neither improved growth (high sublimation temperature), more near-UV absorption (weaker and blue-shifted) nor improved luminescence yield (lower Tb^3+^ lifetime). On the other hand, the main advantage of fluorination is the substantially lower sublimation temperature and a small red-shift of the absorption while the growth and material properties are otherwise very similar to the un-halogenated acid.

**Fig. 10:**
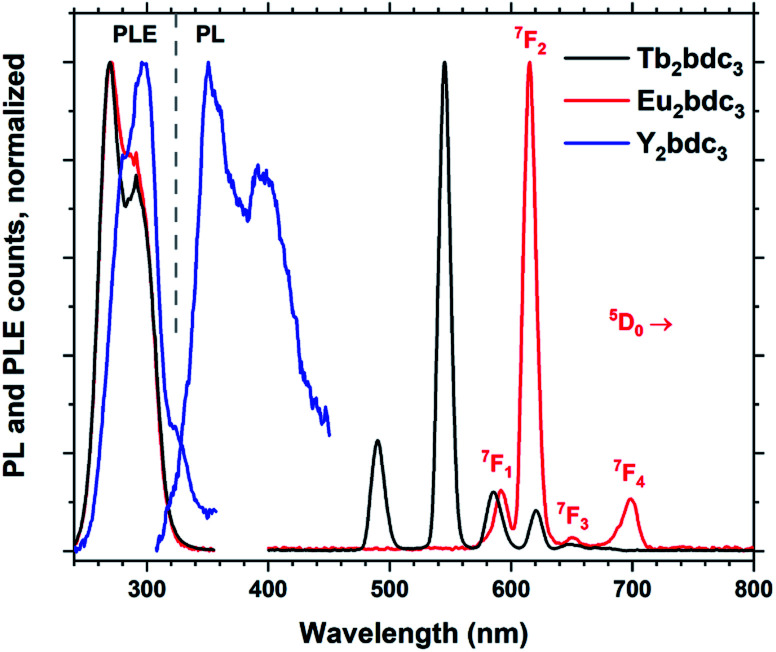
Normalized PLE and PL spectra of Ln_2_bdc_3_, Ln = Tb, Eu and Y. The emission lines of Eu^3+^ are named.

Replacing Tb^3+^ with Eu^3+^ and Y^3+^ results in Ln_2_bdc_3_ films with very different emissions, shown in Fig. 10. The PLE spectra are very similar to each other, although the lighter Y^3+^ shows a small red-shift compared to the heavier Tb^3+^ and Eu^3+^. Both Tb_2_bdc_3_ and Eu_2_bdc_3_ produce their regular Tb^3+^ and Eu^3+^ emission spectra, showing that bdc is an efficient sensitizer for these two lanthanides. Y^3+^ doesn't have a partially filled d or f shell, allowing the sensitizer molecule to emit its own photon. Y_2_bdc_3_ emits in the near-UV, partly extending into the blue range. This gives the material a violet emission colour, relatively weak compared to Tb_2_bdc_3_ and Eu_2_bdc_3_.

### H_2_bdc-NH_2_ and H_2_bdc-OH

These two molecules are interesting from both a chemical and optical perspective. In addition to the two carboxylic acid groups, they have one amine and two hydroxy groups, respectively, which can take part in bonding. Depositions with Tb(thd)_3_ and H_2_bdc-NH_2_ does indeed show a somewhat higher growth rate, while H_2_bdc-OH resulted a fourfold increase in growth rate and strong gradients throughout the gradients. The large increase in growth rate and gradients were reproducible and varying deposition parameters did not result in more homogeneous films. This indicates that the growth chemistry is more complex that for the other sensitizer acids, in particular for H_2_bdc-OH.

**Fig. 11:**
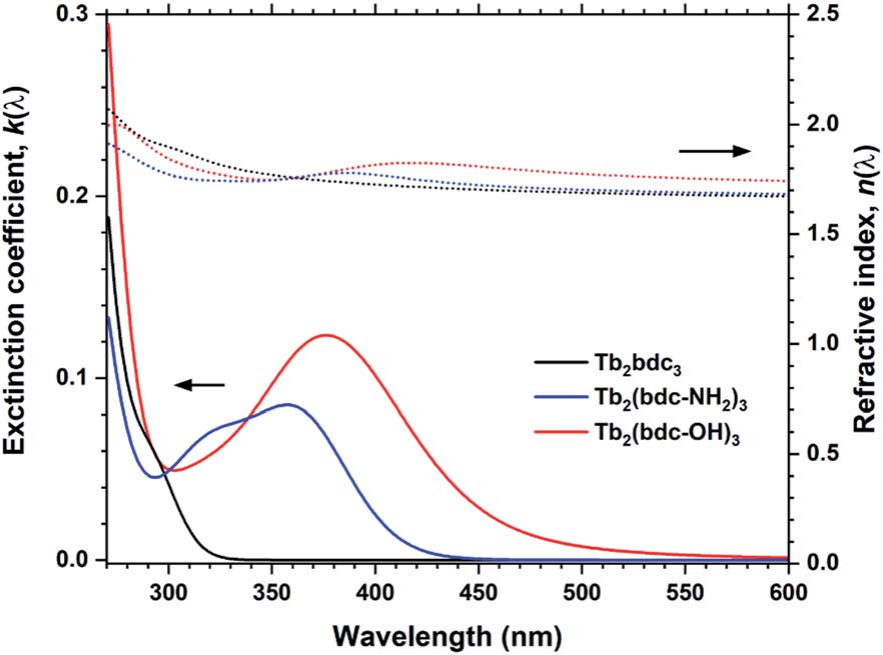
*k*(*λ*) and *n*(*λ*) for Tb_2_bdc_3_, Tb_2_bdc-NH_2_ and Tb_2_bdc-OH films.

These films show large red-shifts compared to Tb_2_bdc_3_, covering the near-UV and even blue range for Tb_2_(bdc-OH)_3_ (Fig. 11). However, all depositions with Tb, Eu and Y with these two acids produced non-luminescent samples. The fact that the Y depositions were quenched indicate that the sensitizer acids are quenched due to how they are packed in the structure. Both acids produce strongly luminescent solutions when dissolved. Crystalline MOF structures with H_2_bdc-NH_2_ and Tb and Eu has been shown to produce sensitized Ln^3+^ emission.^18^ A study by Vogel *et al.* on MOF structures of Y and H_2_bdc-OH show that the exact bonding scheme between Ln^3+^ and the sensitizer acid will have major impacts on the materials optical and luminescent properties.^19^ Thus, although the films produced in our work are non-luminescent, it is likely that control of the resulting structure and molecule packing can produce luminescent films. We have previously shown that control of crystallinity can be achieved both through *in situ* modulation by pulsing other species to modify growth mechanics and ex-situ treatments.^20,21^ However, exploration of how crystallinity and control of growth mechanics can enable luminescent variants is beyond the scope of the current work.

### H_2_bpdc and H_2_ndc

These two biaromatic acids are simpler in terms of not having other functional groups that affect growth. They both produce homogeneous and gradient free films with Y, Tb and Eu, with a slightly larger growth rate than Tb_2_bdc_3_ due to their larger size. Interestingly, Y_2_pbdc_3_ and Y_2_ndc_3_ films show bright violet and blue luminescence from the sensitizer molecule, while absorption covers the near UV range with a tail extending until 400 nm. Thus, due to their good optical properties and even growth, these two sensitizer acids were investigated in more detail.

**Fig. 12:**
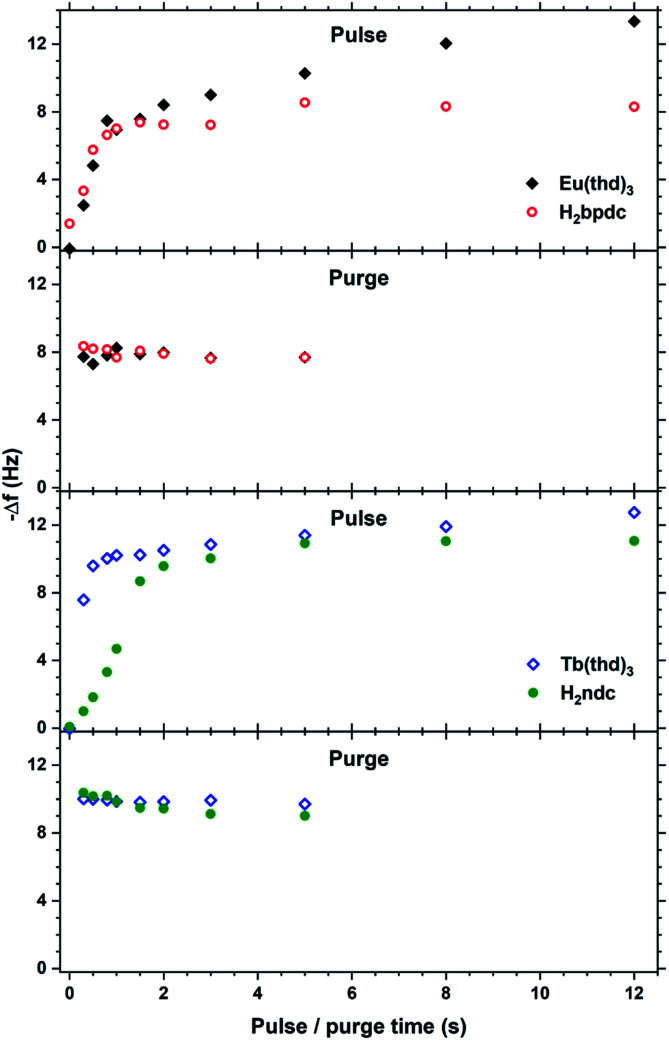
QCM characterization of growth saturation as a function pulse durations for Eu_2_bpdc_3_ and Tb_2_ndc_3_.

The QCM response per cycle Eu_2_bpdc_3_ and Tb_2_ndc_3_ is shown in Fig. 12. Tb_2_ndc_3_ show clear saturation after 1 and 1.5 s Tb(thd)_3_ and H_2_ndc pulses, respectively. Eu_2_bpdc_3_ show strong indication of the same saturation values. However, Eu_2_bpdc_3_ also show a steady increase with increasing Eu(thd)_3_ pulse times longer than 1 s, and some growth at 0 s acid pulses. This is likely due to the higher precursor temperature used for H_2_bpdc. We based the sublimation temperatures on prior sublimations temperatures in ALD-like vacuum conditions. Likely, 250 °C is slightly too warm for this precursor, leading to some precursor vapours seeping into the reaction chamber. This CVD component is quite small for the 2 and 3 s Ln(thd)_3_ and H_2_bpdc pulses used for samples, which was also seen in negligible growth gradients.

**Fig. 13:**
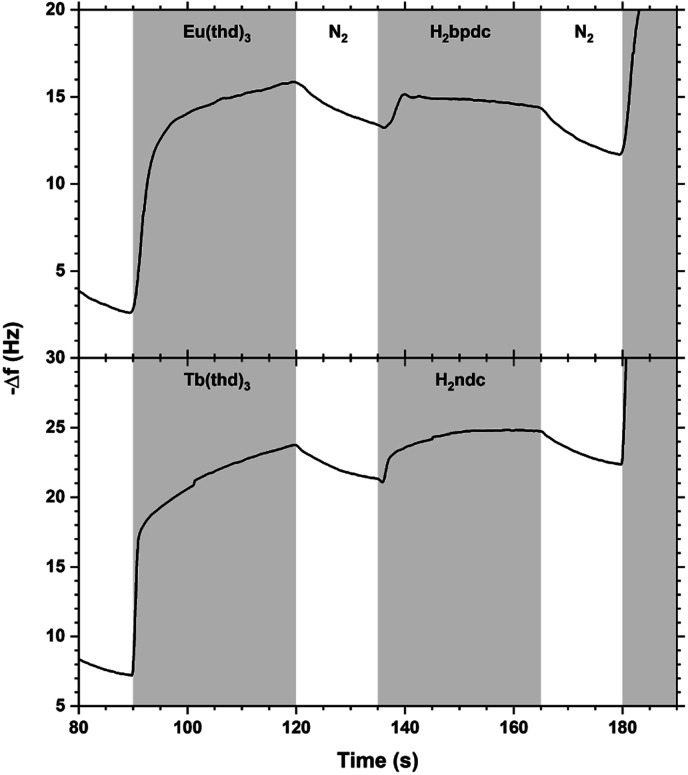
QCM response during a Eu_2_bpdc_3_ and Tb_2_ndc_3_ cycle with long pulse and purge times. Ln(thd)_3_/purge/acid/purge = 30/15/30/15.

The QCM response during a single cycle of Eu_2_bpdc_3_ and Tb_2_ndc_3_ using long pulse and purge times is shown in Fig. 13. As for Tb_2_bdc_3_ in Fig. 3, both Eu_2_bpdc_3_ and Tb_2_ndc_3_ show an initial rapid growth for both Ln(thd)_3_ and acid pulse, indicating surface saturation. Contrary to Tb_2_bdc_3_ which experience a mass decrease during the acid pulse, these two depositions experience a mass gain. This is due to the large H_2_bpdc and H_2_ndc being heavier than Hthd while H_2_bdc is lighter, 242 and 216 g mol^−1^ respectively.

**Fig. 14:**
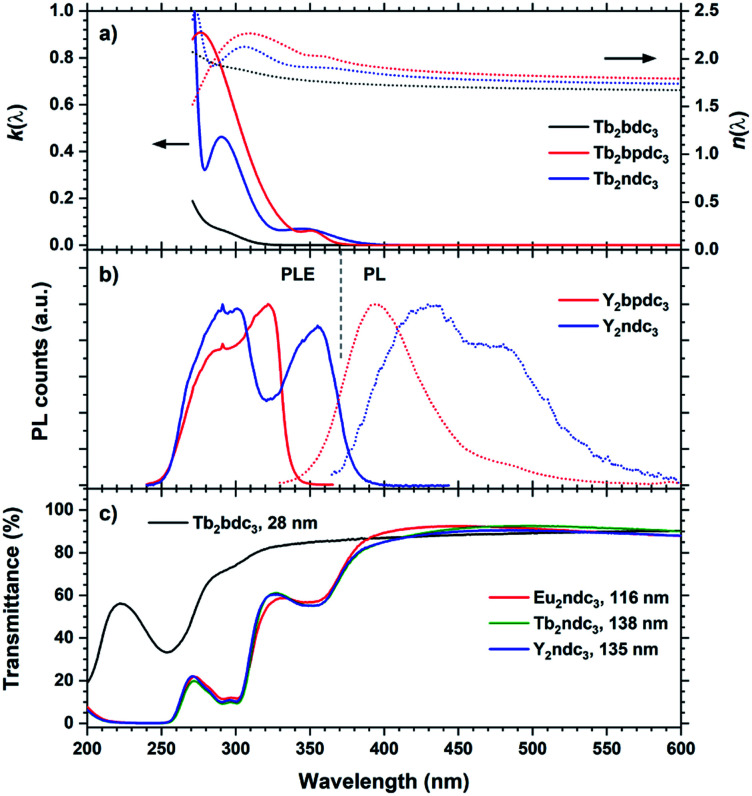
(a) *k*(*λ*) and *n*(*λ*) of Tb_2_bpdc_3_ and Tb_2_ndc_3_. Tb_2_bdc_3_ is included for comparison. (b) PLE and PL of Y_2_bpdc_3_ and Y_2_ndc_3_. *λ*_ex_/*λ*_em_ 320/420 nm and 355/475 nm for Y_2_bpdc_3_ and Y_2_ndc_3_, respectively. (c) Transmission spectra of a selection of thin films from this work, illustrating the strong and near-UV absorption from the organic molecules.

The perhaps most interesting aspects of Ln_2_bpdc_3_ and Ln_2_ndc_3_ films are their optical properties. These films have absorptions covering most of near UV while being almost an order of magnitude stronger than Ln_2_bdc_3_, Ln_2_(bdc-NH_2_)_3_ and Ln_2_(bdc-OH)_3_. Unlike Ln_2_(bdc-NH_2_)_3_ and Ln_2_(bdc-OH)_3_, these films also result in highly luminescent films. Fig. 14 summarizes *k*(*λ*), *n*(*λ*), PLE and optical transmittance of selected films, in addition to the PL spectra originating from the aromatic acids.

Y_2_bpdc_3_ and Y_2_ndc_3_ show strong violet and blue emissions. Their emission spectra are shown in Fig. 14(b). Both consist of broad emission peaks which are normal for aromatic emission. The emission from Y_2_ndc_3_ consist of two distinguishable but overlapping peaks at 430 and 480 nm, while Y_2_bpdc_3_ consist of one major peak at 395 nm and a smaller shoulder at 475 nm. Replacing Y with Tb or Eu fully removed the aromatic emissions, indicating complete energy transfer to these lanthanides in Ln_2_bpdc_3_ and Ln_2_ndc_3_. However, while both Tb and Eu were luminescent in Ln_2_bpdc_3_, only Eu^3+^ was luminescent in Ln_2_ndc_3_. Tb_2_ndc_3_ showed neither aromatic nor Tb emission. The lack of emission from bpdc^2−^ and ndc^2−^ means that there is a complete transfer from these two to something else. ESI Fig. 3† show the emission spectra of Y_2_bpdc_3_ and Y_2_ndc_3_ along with the ground state f–f absorption transitions of Tb^3+^ and Eu^3+^. Both lanthanides show overlapping absorption lines with the emission of both sensitizers. Note that for wavelengths shorter than 400 nm, both lanthanides have dense absorption lines that are excluded in the figure for clarity. The lack of Tb^3+^ emission in Tb_2_ndc_3_ indicated that either Tb^3+^ is fully quenched or that Tb^3+^ never received the energy. In both cases, a possible explanation is a low energy intervalence charge transfer (IVCT) state that can accept the energy from either the sensitizer, lanthanide or both. This IVCT and its quenching on Tb^3+^ is well known in d^0^ metal oxides.^22^ Both Tb^3+^ and Eu^3+^ are redox active (to Tb^4+^ and Eu^2+^, respectively), and so is polyaromatic molecules.^23^ However, a detailed investigation into the quenching mechanisms is beyond the scope of this work.

The Tb and Eu emission spectra were identical to those from Ln_2_bdc_3_ in Fig. 10. In addition, the PLE spectra of Tb and Eu were identical to the PLE spectra of the aromatic emission in Y_2_bpdc_3_ and Y_2_ndc_3_, thus only the PL and PLE spectra of the Y variants are shown in Fig. 14. The modelled *k*(*λ*) and *n*(*λ*) were also identical between lanthanides. Comparing the modelled *k*(*λ*) in (a) and PLE in (b) show that the absorption and excitation peaks matches well for Ln_2_ndc_3_. However, even though both Ln_2_bpdc_3_ and Ln_2_ndc_3_ show similar absorption at 350 nm, this absorption only produces an excitation band in Ln_2_ndc_3_.

In (c) the transmittance spectra on all three Ln_2_ndc_3_ materials are shown alongside Tb_2_bdc_3_ for comparison. All samples are on silica substrates. The absorption bands 350 and 290 nm match well with the modelled *k*(*λ*) and PLE spectre.

## Summary of absorption properties

**Fig. 15:**
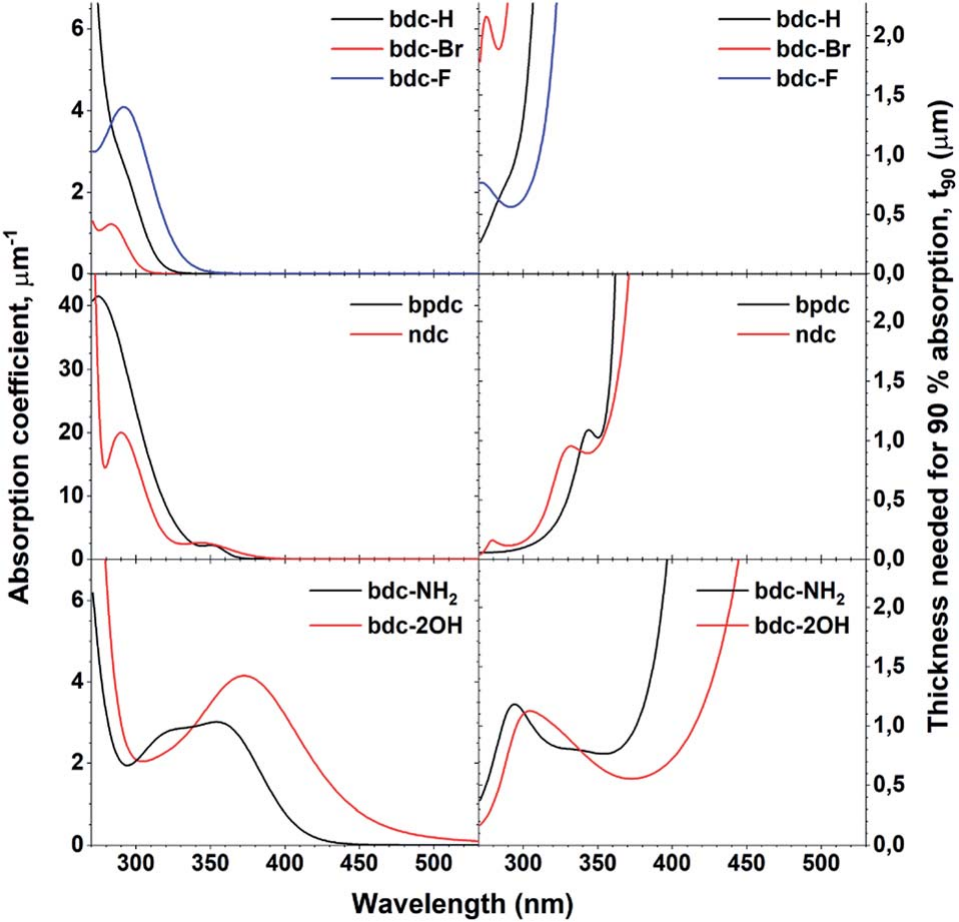
Absorption coefficient of Tb films with all acids, obtained through ellipsometry and the calculated thickness needed for 90% absorption, t_90_. Note the different y-scale on the absorption coefficients for bpdc and ndc.

**Fig. 16:**
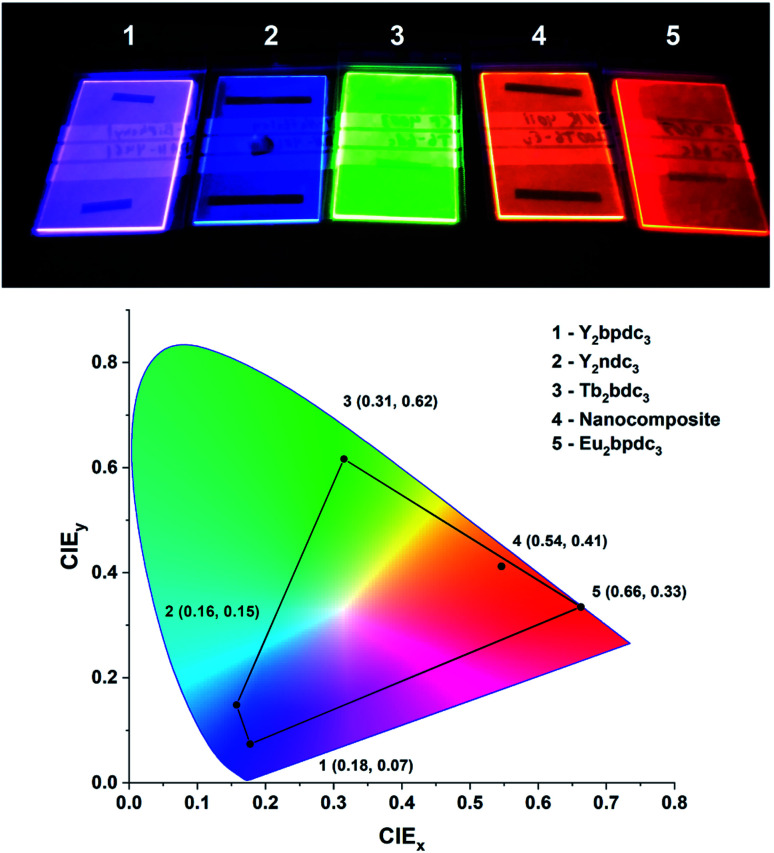
Photo and CIE coordinates of samples from this work under a 254 nm UV lamp. The violet, blue, green and red films as Y_2_bpdc_3_, Y_2_ndc_3_, Tb_2_bdc_3_ and Eu_2_bdc_3_. The orange film is a hybrid-fluoride nanocomposite from another recent work,^8^ showing orange Eu^3+^ emission sensitized by terephthalic acid layers.

One of the defining properties of these mono- and bi-aromatic hybrid films is their strong near-UV absorption. Fig. 15 summarizes the absorption coefficient for all terbium films (note the different y-scale for the two bi-aromatic films). To help visualize how *k*(*λ*) and absorption coefficients manifests as absorption in thin films, the film thickness required for 90% absorption according to Beer–Lambert law is plotted as t_90_. Plotting t_90_ helps understanding how thick films are required for a given absorption. For example, for 90% absorption at 300 nm, films of 100 and 150 nm are sufficient for Ln_2_bpdc_3_ and Ln_2_ndc_3_, while 90% absorption at 375 nm requires 550 nm and 1 μm of Ln_2_(bdc-2OH)_3_ and Ln_2_(bdc-NH_2_)_3_, respectively. The luminescence from these films span the full range from violet to blue, green and red as shown in Fig. 16 along with the films colour coordinates.

## Conclusion

In this work we have deposited hybrid films of lanthanides with 9 different mono- and bi-aromatic di-carboxylic acids, and explored their optical and luminescent properties. The terbium–terephthalic acid system has been used as a base and is explored in more detail, including film growth and air stability. Yttrium has been used as an optically passive cation so that the luminescence properties of the aromatic molecule itself can be investigated. The yttrium films show that the two bi-aromatic acids produce films with bright violet and blue luminescence. Yet with Tb and Eu all bi-aromatic films are non-luminescent except Eu_2_ndc_3_. The bi-aromatic films show by far the strongest absorption strength of all the acids. The two acids with added functional groups on the other hand, namely bdc-2OH and bdc-NH_2_, show the largest red-shift in absorption and even reaches slightly into the blue range for bdc-2OH. In conclusion, the optical properties of hybrid materials made from lanthanides and aromatic di-carboxylic acids show a large variation in both absorption range and strength while their luminescence spans violet, blue, green and red.

## Author contributions

Hansen: conceptualization, data curation, formal analysis, supervision, writing – original draft. Svendsen: investigation, formal analysis, writing – review & editing. Nesteng: investigation, formal analysis, writing – review & editing. Nilsen: data curation, formal analysis, supervision, writing – review & editing, project administration, funding acquisition.

## Conflicts of interest

There is no conflict of interest to report.

## Supplementary Material

RA-012-D2RA03360G-s001
