# Supplementary material for: Aromatic sensitizers in luminescent hybrid films
Source: RSC Adv. 2022 Jun 20;12(28):18063–71. doi: 10.1039/d2ra03360g (PMC9207708; doi:10.1039/d2ra03360g)
Supplement: RA-012-D2RA03360G-s001 [file RA-012-D2RA03360G-s001.pdf]

Electronic Supplementary Material (ESI) for RSC Advances.
This journal is © The Royal Society of Chemistry 2022

Supplementary information

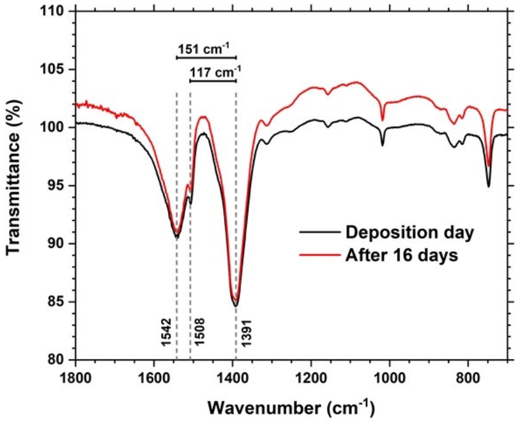

Supplementary figure 1: Zoomed in FTIR spectra.

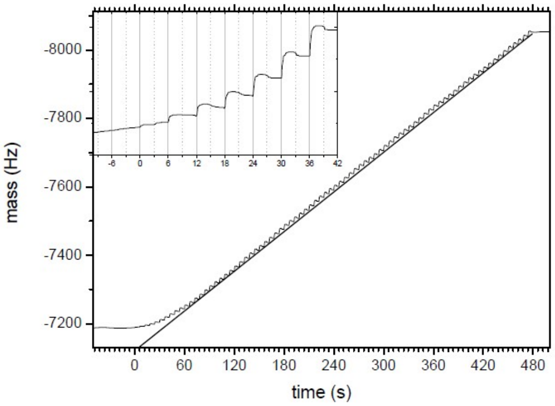

Supplementary figure 2: Detailed view of the first Tb_2bdc_3 cycles in a QCM experiment, also showing linear growth after the initial growth.

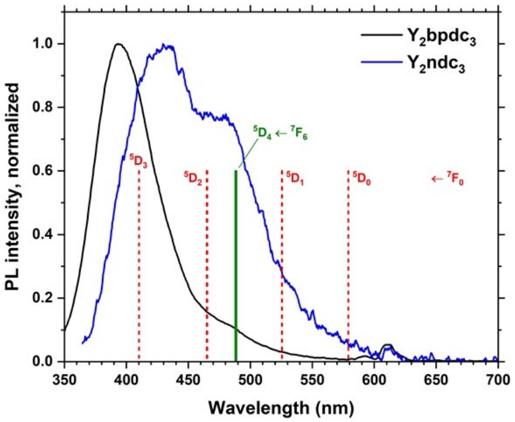

Supplementary figure 3: Emission from $bpdc^{2-}$ and ndc^{2-} in Y_2bpdc_3 and Y_2ndc_3, and the position of the f-f absorption lines of Tb^{3+} (solid line) and Eu^{3+} (staplede line) stemming from the ground state.
